# Haptoglobin inhibits phospholipid transfer protein activity in hyperlipidemic human plasma

**DOI:** 10.1186/1476-511X-8-27

**Published:** 2009-07-23

**Authors:** Ryan J Henderson, Kishor M Wasan, Carlos G Leon

**Affiliations:** 1 Division of Pharmaceutics and Biopharmaceutics, Faculty of Pharmaceutical Sciences, The University of British Columbia, Vancouver, British Columbia, V6T 1Z3, Canada

## Abstract

**Background:**

Haptoglobin is a plasma protein that scavenges haemoglobin during haemolysis. Phospholipid Transfer Protein (PLTP) transfers lipids from Low Density Lipoproteins (LDL) to High Density Lipoproteins (HDL). PLTP is involved in the pathogenesis of atherosclerosis which causes coronary artery disease, the leading cause of death in North America. It has been shown that Apolipoprotein-A1 (Apo-A1) binds and regulates PLTP activity. Haptoglobin can also bind to Apo-A1, affecting the ability of Apo-A1 to induce enzymatic activities. Thus we hypothesize that haptoglobin inhibits PLTP activity. This work tested the effect of Haptoglobin and Apo-A1 addition on PLTP activity in human plasma samples. The results will contribute to our understanding of the role of haptoglobin on modulating reverse cholesterol transport.

**Results:**

We analyzed the PLTP activity and Apo-A1 and Haptoglobin content in six hyperlipidemic and six normolipidemic plasmas. We found that Apo-A1 levels are proportional to PLTP activity in hyperlipidemic (R^2 ^= 0.66, p < 0.05) but not in normolipidemic human plasma. Haptoglobin levels and PLTP activity are inversely proportional in hyperlipidemic plasmas (R^2 ^= 0.57, p > 0.05). When the PLTP activity was graphed versus the Hp/Apo-A1 ratio in hyperlipidemic plasma there was a significant correlation (R^2 ^= 0.69, p < 0.05) suggesting that PLTP activity is affected by the combined effect of Apo-A1 and haptoglobin. When haptoglobin was added to individual hyperlipidemic plasma samples there was a dose dependent decrease in PLTP activity. In these samples we also found a negative correlation (-0.59, p < 0.05) between PLTP activity and Hp/Apo-A1. When we added an amount of haptoglobin equivalent to 100% of the basal levels, we found a 64 ± 23% decrease (p < 0.05) in PLTP activity compared to basal PLTP activity. We tested the hypothesis that additional Apo-A1 would induce PLTP activity. Interestingly we found a dose dependent decrease in PLTP activity upon Apo-A1 addition. When both Apo-A1 and Hpt were added to the plasma samples there was no further reduction in PLTP activity suggesting that they act through a common pathway.

**Conclusion:**

These findings suggest an inhibitory effect of Haptoglobin over PLTP activity in hyperlipidemic plasma that may contribute to the regulation of reverse cholesterol transport.

## Background

Haptoglobin is an acute phase protein that scavenges haemoglobin released into the circulation [1]. Haptoglobin, the plasma protein with highest binding affinity to haemoglobin, is mainly expressed in the liver [2]. It plays an anti-oxidant role by binding free haemoglobin and forming a complex that is taken up by hepatocytes and macrophages [3]. The human haptoglobin gene encompasses three alleles: Hp1F, Hp1S and Hp2 [4]. The Hp2 allele is the fusion product of the Hp1F and Hp1S alleles. Haptoglobin presents as a dimer of two of these alleles which binds to one haemoglobin dimer [2]. Haptoglobin expression is induced several fold in the event of inflammation triggered by infection, injury or cancer development [1,5]. Haptoglobin has been shown to play an antioxidant/anti-inflammatory role, to contribute to neutrophil activation [6], to maintain reverse cholesterol transport [7] and to modulate the inhibition of cyclooxygenase and lipooxygenase [8], amongst other functions. In particular, haptoglobin has been shown to inhibit Lecithin-Cholesterol Acyltransferase (LCAT) in human ovarian follicular fluid [9]. LCAT is involved in the removal of cholesterol excess from peripheral tissues [10]. LCAT transfers an acyl chain from high density lipoprotein (HDL) lecithin to cellular cholesterol. This activity is stimulated by the presence of Apo-A1, the main protein constituent of HDL. Balestrieri et al [9] demonstrated that LCAT activity is negatively correlated with the Hp/Apo-A1 ratio in human follicular fluid. The mechanism of action of haptoglobin inhibition of LCAT activity has been described [11]. The binding site of Haptoglobin on Apo-A1 has been mapped and it was demonstrated that the interaction of haptoglobin to Apo-A1 is independent to the binding of haptoglobin and haemoglobin. A peptide designed based on the sequence in Apo-A1 that putatively interacts with Haptoglobin was shown to restore LCAT activity inhibited by Hp demonstrating that the Apo-A1-Hp interaction is responsible for the inhibition of LCAT activity. Based on this evidence it has been speculated that haptoglobin may play a role in the inhibition of reverse cholesterol transport.

In the present study we investigated the effect of haptoglobin on the activity of another enzyme involved in reverse cholesterol transport, phospholipid transfer protein (PLTP). PLTP is a plasma protein that transfers phospholipids from triglyceride-rich lipoproteins such as very low-density lipoproteins (VLDL) and low-density lipoproteins (LDL) to high density lipoproteins (HDL) [12,13]. PLTP occurs in plasma as two main forms: a high activity PLTP (HA-PLTP) and a low activity PLTP (LA-PLTP). HA-PLTP is associated with the majority of plasma PLTP activity. PLTP activity has been shown to be affected by its association to Apo-A1 [14,15]. There is increasing evidence supporting the role of PLTP on atherosclerosis development [16]. Moerland et al., [17] showed in a transgenic mouse model of PLTP expression that an acutely increased PLTP expression resulted in a highly atherogenic lipoprotein profile. Shelly et al., [18] found that the phospholipid transfer protein deficiency ameliorated diet-induced hypercholesterolemia and inflammation in mice. There is evidence that even a 10% reduction on PLTP activity can lead to a significant reduction of atherosclerosis progression [19], highlighting the role of PLTP on the development of cardiovascular disease.

In the present study we hypothesize that haptoglobin inhibits PLTP activity. This is based on the fact that PLTP activity is dependent on its binding to Apo-A1 [15] and that haptoglobin has been shown to bind Apo-A1 [11] and to inhibit LCAT activity [9]. This work will further contribute to our understanding of the role of haptoglobin on modulating reverse cholesterol transport as well as the development of atherosclerosis.

## Results

### Haptoglobin and Apolipoprotein A1 levels in normolipidemic and hyperlipidemic human plasma

Haptoglobin and Apo-A1 levels were determined for each one of the plasma samples (Table 1). When the Hp levels were compared between the two groups (hyperlipidemic vs. normolipidemic) no difference was found. Likewise, when the levels of Apo-A1 were compared amongst the two groups they were not different.

**Table 1:** Apolipoprotein A1 (μg/mL) and Haptoglobin (μg/mL) levels in hyperlipidemic (H1-H6) and normolipidemic (N1-N6) plasmas used in this study (mean ± SD).

Plasma #	Apo-A1 (μg/mL)	Haptoglobin (μg/mL)
H1	3.9 ± 0.1	5897.7 ± 527
H2	23.2 ± 1.9	5240.4 ± 110
H3	275.7 ± 13	1245.7 ± 28
H4	265 ± 26	4579.9 ± 259
H5	185.8 ± 9	318.6 ± 14
H6	167.3 ± 7	3891.1 ± 231
		
N1	161.2 ± 4.9	500.5 ± 41
N2	186.2 ± 16	6104.2 ± 134
N3	201 ± 2.1	1480 ± 99
N4	54.8 ± 3	4126.5 ± 124
N5	269.9 ± 34	1322.7 ± 48
N6	337.7 ± 28	3125.5 ± 48

Apo-A1 and haptoglobin were measured as described in Materials and Methods.

### Inverse association between PLTP activity and haptoglobin levels in hyperlipidemic plasma

**Figure 1:**
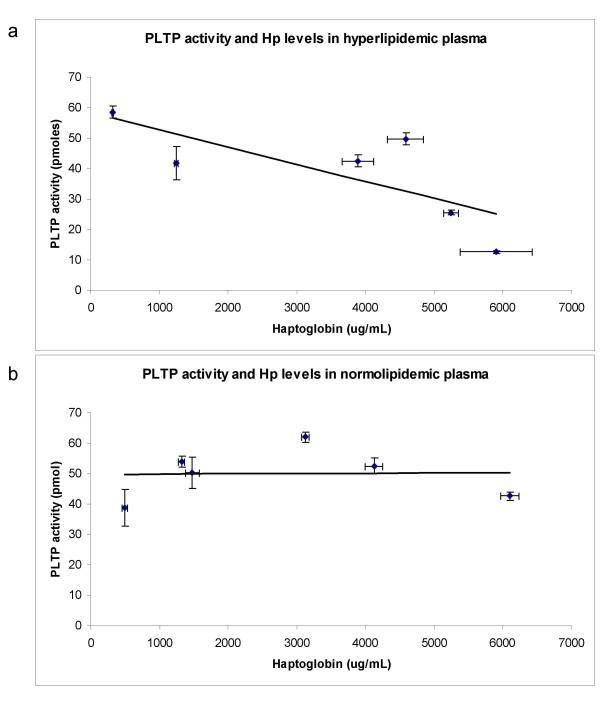
**a. Association between PLTP activity and haptoglobin levels in hyperlipidemic plasma (R^2 ^= 0.5733, n = 6)**. PLTP activity after 60 min was determined as described in Materials and Methods. Haptoglobin levels were determined using a two site Human Haptoglobin ELISA kit. Each plasma sample was analyzed in two independent experiments, with at least two replicates per experiment. **b**. Association between PLTP activity and haptoglobin levels in normolipidemic plasma (R^2 ^= 0.0007, n = 6). PLTP activity after 60 min was determined as described in Materials and Methods. Haptoglobin levels were determined using a two site Human Haptoglobin ELISA kit. Each plasma sample was analyzed in two independent experiments, with at least two replicates per experiment.

We determined the PLTP activity as described elsewhere [20]. When we graphed the PLTP activity vs. the haptoglobin levels we found a trend of a correlation (Figure 1a R^2 ^= 0.57, Correlation coefficient -0.75) albeit not significant (p = 0.08). In the case of the normolipidemic plasma there was no indication of a correlation (Figure 1b).

### Direct association between PLTP activity and Apo-A1 levels in hyperlipidemic plasma. Correlation between the PLTP activity and the Hp/Apo-A1 ratio in hyperlipidemic plasma

**Figure 2:**
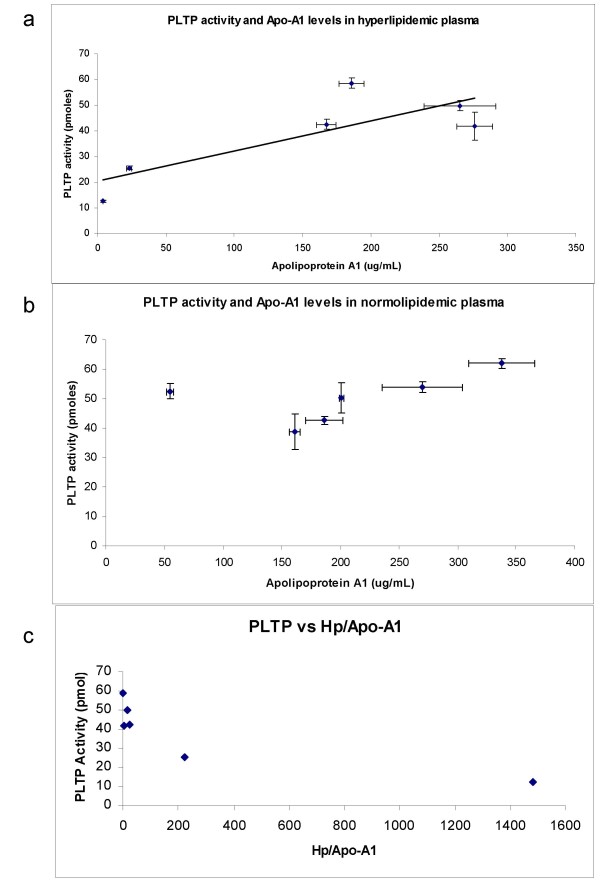
**a. Association between Apolipoprotein A1 levels and PLTP activity (60 min) in hyperlipidemic plasma**. Apolipoprotein levels were determined using an EIA kit as outlined in Materials and Methods. Each plasma sample was analyzed in two independent experiments, with at least two replicates per experiment. The graph depicts the mean ± standard deviation (R^2 ^= 0.6635, n = 6, p < 0.05). **b**. Association between Apolipoprotein A1 levels and PLTP activity (60 min) in normolipidemic plasma (R^2 ^= 0.2732, n = 6, p > 0.05). **c**. Association between PLTP activity (60 min) and Hp/Apo-A1 ratio in hyperlipidemic plasma (R^2 ^= 0.69, n = 6, p < 0.05).

When we compared the PLTP activity vs. the Apo-A1 levels we found a positive correlation between these two variables (Figure 2a, R^2 ^= 0.66, Correlation coefficient 0.81, p < 0.05) in hyperlipidemic plasma but not in normolipidemic plasma (Figure 2b).

Based on the model of LCAT inhibition by haptoglobin, we determined the relationship between PLTP activity and Ht/Apo-A1 ratio (Figure 2c) in hyperlipidemic plasma. A negative linear correlation was found (R^2 ^= 0.69, p < 0.05) suggesting an inhibitory role of haptoglobin on PLTP activity in this group of plasmas. When a semi logarithmic non linear regression was used, we obtained a higher correlation (R^2 ^= 0.998) than with the linear model.

### Inhibition of PLTP activity by the addition of increasing concentrations of haptoglobin

**Figure 3:**
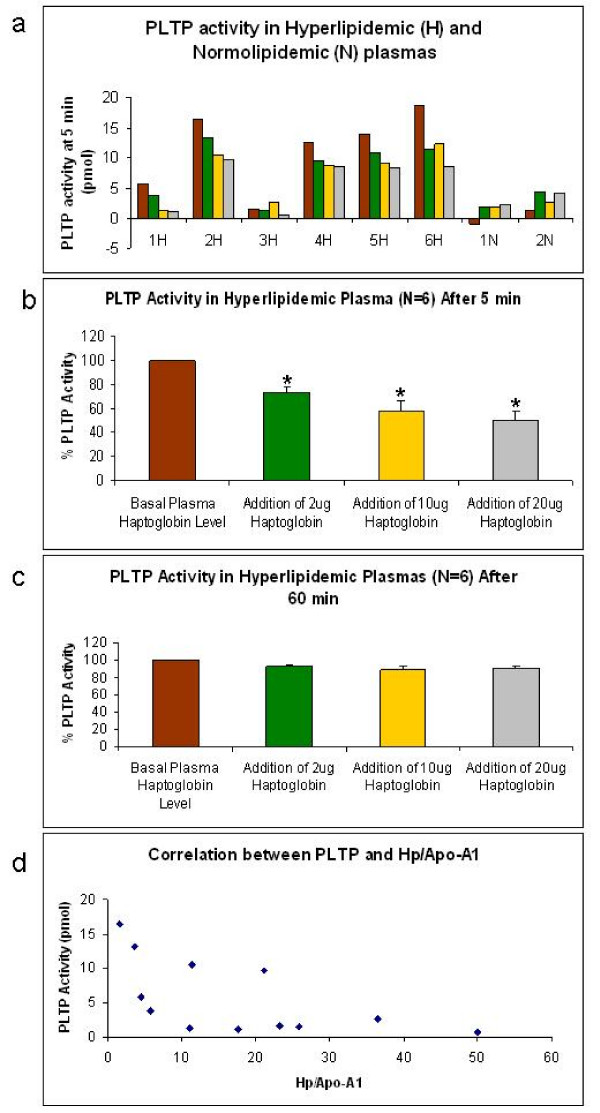
**a. Effect of haptoglobin addition (2, 10 and 20 μg per sample) on PLTP activity (5 min) of individual hyperlipidemic (H, n = 6) and normolipidemic (N, n = 2) plasma samples**. From left to right: control, addition of 2 μg, 10 μg and 20 μg of Hp, respectively. One of two representative experiments with duplicate measurements per treatment. **b**. Effect of haptoglobin addition (2, 10 and 20 μg per sample) on PLTP activity (5 min) in hyperlipidemic plasma as a percentage of basal PLTP activity. Each plasma sample was analyzed in two independent experiments, with at least two replicates per experiment. The graph depicts the mean ± standard deviation of each group of six hyperlipidemic plasma (n = 6, p < 0.05). **c**. Ibidem, except that PLTP activity was analyzed after 60 min. **d**. Correlation between PLTP activity and Hp/Apo-A1 ratio in hyperlipidemic plasma samples with added haptoglobin (n = 11, Correlation coefficient -0.679, p < 0.05).

**Figure 4:**
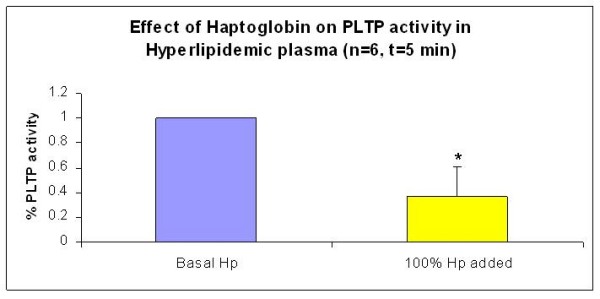
**Effect of Haptoglobin addition on PLTP activity (5 min) in hyperlipidemic plasma**. The equivalent of 100% of basal haptoglobin levels was added to each plasma sample and PLTP activity was measured as described previously (n = 6, p < 0.05).

To further examine the inhibition of PLTP activity, increasing amounts of haptoglobin were added to our set of plasma samples (Figure 3a). Five out of six hyperlipidemic plasma showed a decreased PLTP activity with an increasing dose of added haptoglobin, irrespective of the basal PLTP activity. On the other hand, the three normolipidemic plasma samples analyzed didn't show any clear trend which is in agreement with our previous results which do not indicate a role of haptoglobin inhibition in this group. We further analyzed the data expressing it as percentage inhibition of PLTP activity and found a significant decrease on PLTP activity in the hyperlipidemic plasma after 5 min of haptoglobin addition (Figure 3b). This effect was reduced after 60 min of haptoglobin addition (Figure 3c). When we analyzed the PLTP activity vs. the Hp/Apo-A1 ratio, we also found a negative correlation (Figure 3d, -0.697, p = 0.0217 and n = 11). Based on the initial amount of haptoglobin in each plasma sample, we added this specific amount of haptoglobin to each sample. The effect was a 64% reduction in PLTP activity compared to untreated controls (Figure 4, p < 0.05). This fact further supported an inhibitory role of haptoglobin over PLTP activity *in vitro*.

### Inhibition of PLTP activity by the addition of increasing concentrations of haptoglobin

**Figure 5:**
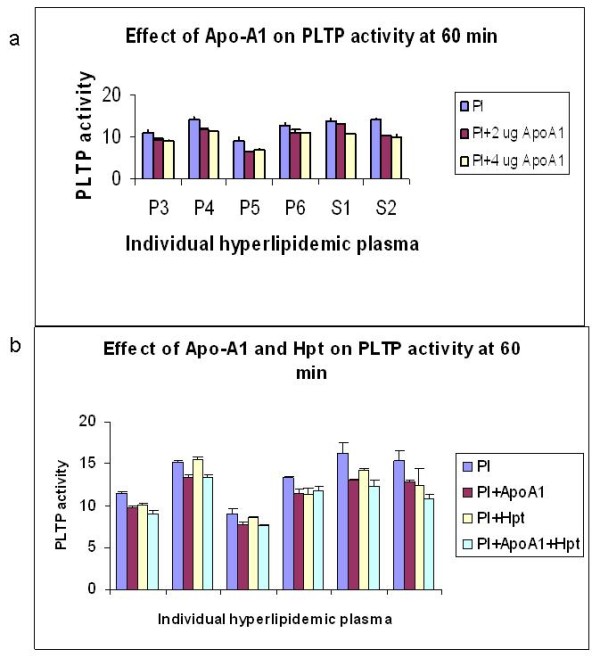
**a. Effect of Apo-A1 addition to the rate of PLTP activity within the first 60 minutes of reaction in hyperlipidemic plasma**. Two and four micrograms of Apo-A1 were added to the plasma samples and PLTP activity was measured within the first two and 60 minutes to calculate the rate of activity (nmoles product/min). One of two representative experiments. **b**. Effect of Apo-A1 and Haptoglobin addition to the rate of PLTP activity within the first 60 minutes of reaction in hyperlipidemic plasma. Two micrograms of Apo-A1 and/or the equivalent of 100% of basal haptoglobin were added to the plasma (Pl: plasma alone, Pl+Apo-A1: plasma plus Apo-A1, Pl+Hp: plasma plus haptoglobin and Pl+Apo-A1+Hp: plasma plus Apo-A1 plus Haptoglobin) and PLTP activity was measured within the first two and 60 minutes to calculate the rate of activity (nmoles product/min). One of three representative experiments.

We further explored the possible role of Apo-A1 on the haptoglobin inhibition of PLTP activity. Interestingly, we found that Apo-A1 inhibited the PLTP activity in the six hyperlipidemic plasmas (Figure 5a). We also confirmed the Hp inhibitory effect on PLTP. When both Apo-A1 and Hp were added, no additive effect was observed (Figure 5b) suggesting that their inhibitory effect occurs through a common mechanism.

## Discussion

Haptoglobin genotype has been shown to regulate reverse cholesterol transport in diabetes *in vitro *and *in vivo *[21]. It has been proposed that an enhanced oxidative modification of serum lipoproteins (LDL and HDL) in individuals with the Hp2 genotype is an important determinant of accelerated atherosclerosis in these individuals [22]. Interestingly, PLTP has been shown to efflux cholesterol Apo-A1 in murine macrophages [23].

Another mechanism by which haptoglobin may regulate reverse cholesterol transport is by inhibiting LCAT [9]. Since this inhibition is mediated through the Hp-Apo-A1 interaction [11], we propose that other enzymatic activities regulated by Apo-A1 may be affected by haptoglobin levels. In particular, we are interested in PLTP which is an important enzyme involved in reverse cholesterol transport [13] and its activity has been shown to be dependent on its association with Apo-A1 [15]. The positive correlation between PLTP activity and Apo-A1 levels in hyperlipidemic plasma persisted. However, we didn't observe this correlation in normolipidemic patients. A positive correlation was found between PLTP activity and Apo-A1 in plasma from patients with type 1 diabetes [24] which is consistent with our observations. These authors had previously demonstrated that patients with type 1 diabetes have a significantly elevated PLTP activity and that this activity is correlated with HDL levels [25]. One of the differences between the two plasma groups that we used was the HDL content. We found a negative correlation (p = 0.018) between PLTP activity and HDL levels. This correlation was specific to HDL as it was not found with total cholesterol and triglycerides. Colhoun et al., [24] showed differences between the correlation of PLTP activity and HDL particle size. PLTP activity negatively correlated with small HDL while it positively correlated with large HDL. Soro et al., [26] also showed a negative correlation between HDL2 and PLTP activity. Since there is an association between reduced HDL particle size and hyperlipidemia [27], it is possible that in our hyperlipidemic patient group there is a higher small HDL/large HDL ratio than in normolipidemic controls and this contributes to a negative correlation between PLTP activity and HDL.

One of the limitations of our study is the sample size. However the correlations that we observed in the basal state were maintained even when exogenous Hp and Apo-A1 were added to the system. Nevertheless, Salvatore et al., [28] found a correlation between the cholesteryl ester/cholesterol ratio (a measure of LCAT activity) and Hp/[Apo E +Apo-A1] ratio in a small number of multiple sclerosis patients (n = 9).

When we compared haptoglobin levels with PLTP activity, the correlation was insignificant (p = 0.08). However, when we changed our analysis to compare PLTP activity with Hp/Apo-A1 ratio, we did find a negative correlation suggesting a) that haptoglobin may inhibit PLTP activity and b) that Apo-A1 levels may affect this inhibitory interaction.

We further confirmed the Hp inhibitory effect by adding exogenous haptoglobin to the plasma samples. In hyperlipidemic plasma, PLTP activity was inhibited by haptoglobin addition in a dose-dependent way. This effect was not seen in normolipidemic plasma. This effect could also be related to the differences in low activity and high activity PLTP in hyperlipidemic and normolipidemic plasma and the differential association of Apo-A1 to these two forms of PLTP [14]. The fact that PLTP activity is negatively correlated with the Hp/Apo-A1 ratio in hyperlipidemic plasma has not been previously reported.

## Conclusion

PLTP activity was inhibited by Haptoglobin and Apo-A1 addition. Haptoglobin, HDL and PLTP activity correlation data suggests the potential to use haptoglobin as a biomarker for the development of atherosclerosis as well as a tool to understand the role of PLTP activity and haptoglobin levels in reverse cholesterol and atherosclerosis.

## Materials and methods

### Chemicals

PLTP Activity Assay Kit's were obtained from Roar Biomedical (New York, NY, USA). Purified Hpt (at least 95% pure by SDS-PAGE) was purchased from Calbiochem (San Diego, CA). Apolipoprotein A1 was purchased from Sigma-Aldrich (St Louis, MI).

### Plasma Samples

Twelve different human plasma samples (purchased from Bioreclamation [East Meadow, NY, USA]) were obtained from donors representing both normolipidemic plasma (N = 6) and hyperlipidemic plasma (N = 6) based on the standards set by the Ministry of Health and Welfare of Japan (cholesterol <220 mg/dl and triglycerides <150 mg/dl) [29]. The cholesterol and triglyceride content of each one of these twelve samples has been reported previously [20].

### Haptoglobin determination

Haptoglobin levels were determined using a two site Human Haptoglobin ELISA kit from ICL (Newberg, OR) as per the manufacturer instructions. Briefly, the control, standard and patient samples were added to the wells which had previously adsorbed the anti-Hp antibodies. The unbound proteins were removed by washing, and then anti-Hp antibodies conjugated to horseradish peroxidase were added. These enzyme-labeled antibodies form complexes with the previously bound plasma Hp. Following washings, a chromogenic substrate was added and absorbance was read at 450 nm. The concentration of Hp was determined using a standard curve of purified Hp.

### Apo-A1 determination

The human Apo-A1 EIA kit was purchased from Cayman Chemical Company (Ann Arbor, MI). Briefly each well of the plate provided with the kit was coated with an Apo-A1 specific antibody. When the samples and controls were added to the wells any Apo-A1 would bind to these antibodies. After washings a new anti Apo-A1 antibody was added to detect the captured Apo-A1. Washings were followed by the addition of horseradish peroxidase conjugate that will recognize the complex. Upon washings and addition of a chromogenic substrate, the reaction was stopped with acid and absorbance was read at 450 nm. The intensity of the color is proportional to the concentration of Apo-A1 which was determined using a standard curve.

### PLTP Assay

Each plasma sample was tested for PLTP activity using an activity assay that measures *in vitro *phospholipid transfer activity (Roar Biomedical, New York, NY). The PLTP assay was carried as per manufacturer instructions as described previously [20]. Initially, the PLTP activity test was studied for different concentrations of plasma protein and it was decided to be 25 μg as the PLTP activity was linear in this range. PLTP activity was determined for plasma samples (25 μg). Controls were added in the form of a picomol standard to quantify PLTP activity and a blank control. Plates also measured plasma activity as a single entity in the PLTP kit. Measurements were taken for 6 different plasma samples in the hyperlipidemic range and 6 different plasma samples in the normolipidemic range. These samples were done in duplicate on each 96-well test plate and each test plate was repeated at least twice for each plasma sample.

### Statistical Analysis

The groups tested in this study were compared against each other by applying a repeated measure analysis of variance (ANOVA) test and blocking results in set plasmas to account for base PLTP activity variance. Statistical differences in the data was considered significant if the p value found was < 0.05. Data added for each plasma measure consisted of an N = 6 of which each experiment had at least a replicate value of 2.

The strength and direction of a linear relationship between two random variables was measured by the Pearson's coefficient of correlation as determined using SigmaStat™.

## Abbreviations

Hp: Haptoglobin; PLTP: Phospholipid transfer protein; Apo-A1: Apolipoprotein A1; HDL: High density lipoprotein; LDL: Low density Lipoprotein.

## Competing interests

The authors declare that they have no competing interests.

## Authors' contributions

RJH conceived the study and carried out the experiments and revised the manuscript. KMW participated in the design of the study, data analysis and helped to draft the manuscript. CGL participated in the design and coordination of the study, and drafted the manuscript. All authors read and approved the final manuscript.
